# Pathogenic Mutations Differentially Regulate Cell-to-Cell Transmission of α-Synuclein

**DOI:** 10.3389/fncel.2020.00159

**Published:** 2020-06-12

**Authors:** Yuan Guan, Xiaofang Zhao, Fengwei Liu, Shuxin Yan, Yalong Wang, Cuilian Du, Xiuyu Cui, Rena Li, Claire Xi Zhang

**Affiliations:** ^1^Beijing Institute of Brain Disorders, Laboratory of Brain Disorders, Ministry of Science and Technology, Collaborative Innovation Center for Brain Disorders, Capital Medical University, Beijing, China; ^2^Department of Anesthesiology, Beijing Huaxin Hospital, First Hospital of Tsinghua University, Beijing, China; ^3^Institute of Clinical Neuroimmunology, University Hospital and Biomedical Center, Ludwig-Maximilians University Munich, Munich, Germany; ^4^Beijing Key Laboratory of Mental Disorders, Beijing Anding Hospital and Beijing Institute of Brain Disorders, Capital Medical University, Beijing, China

**Keywords:** Parkinson’s disease, α-synuclein, cell-to-cell transmission, secretion, uptake, toxicity, aggregation

## Abstract

Recent studies suggest that the cell-to-cell spread of pathological α-synuclein (α-syn) plays important roles in the development of Parkinson’s disease (PD). PD patients who carry α-syn gene mutations often have an earlier onset and more severe clinical symptoms and pathology than sporadic PD cases who carry the wild-type (WT) α-syn gene. However, the molecular mechanism by which α-syn gene mutations promote PD remains unclear. Here, we hypothesized that pathogenic mutations facilitate the intercellular transfer and cytotoxicity of α-syn, favoring an early disease onset and faster progression. We investigated the effects of eight known pathogenic mutations in human α-syn (A18T, A29S, A30P, E46K, H50Q, G51D, A53E, and A53T) on its pathological transmission in terms of secretion, aggregation, intracellular level, cytotoxicity, seeding, and induction of neuroinflammation in SH-SY5Y neuroblastoma cells, cultured rat neurons, and microglia, and the rat substantia nigra pars compacta. We found that 2 of the 8 mutations (H50Q and A53T) significantly increased α-syn secretion while 6 mutations (A18T, A29S, A30P, G51D, A53E, and E46K) tended to enhance it. *In vitro*α-syn aggregation experiments showed that H50Q promoted while G51D delayed aggregation most strongly. Interestingly, 3 mutations (E46K, H50Q, and G51D) greatly increased the intracellular α-syn level when cultured cells were treated with preformed α-syn fibrils (PFFs) compared with the WT, while the other 5 had no effect. We also demonstrated that H50Q, G51D, and A53T PFFs, but not E46K PFFs, efficiently seeded *in vivo* and acutely induced neuroinflammation in rat substantia nigra pars compacta. Our data indicate that pathogenic mutations augment the prion-like spread of α-syn at different steps and blockade of this pathogenic propagation may serve as a promising therapeutic intervention for PD.

## Introduction

α-Synuclein (α-syn) was the first causative gene of Parkinson’s disease (PD) to be discovered (Polymeropoulos et al., 1997). Abnormally aggregated α-syn protein is the main component of Lewy bodies and Lewy neurites, which are classical markers of the pathology of PD (Spillantini et al., 1997). Genetic studies indicate that single-nucleotide polymorphisms in the SNCA gene are strongly associated with familial inherited PD (Pals et al., 2004; Pankratz et al., 2009; Rajput et al., 2009). Eight point mutations have been identified to be pathogenically associated with PD: A18T, A29S, A30P, E46K, H50Q, G51D, A53E, and A53T (Polymeropoulos et al., 1997; Kruger et al., 1998; Zarranz et al., 2004; Appel-Cresswell et al., 2013; Hoffman-Zacharska et al., 2013; Kiely et al., 2013; Pasanen et al., 2014). The onset time, clinical symptoms, and pathology of these mutations are quite distinctive (Fujioka et al., 2014). A18T, A29S, and A30P are associated with a relatively typical PD phenotype and mild clinical manifestations (Kruger et al., 2001; Hoffman-Zacharska et al., 2013), but patients with E46K, H50Q, G51D, A53E, or A53T have a severe and rather rapidly progressive clinical phenotype, involving cognitive impairment, psychiatric disturbance, hallucination, autonomic dysfunction, myoclonus, and epilepsy with pyramidal signs (Zarranz et al., 2004; Appel-Cresswell et al., 2013; Kiely et al., 2013; Lesage et al., 2013; Proukakis et al., 2013; Pasanen et al., 2014). Among these, G51D and A53T are especially notable for early onset and severe symptoms. Much research has been carried out on these mutations as they provide valuable tools to understand the pathogenesis of PD. However, the mechanisms underlying the clinical phenotypes remain elusive.

Previously, it was thought that α-syn misfolding and intracellular aggregation play a central pathogenic role in synucleinopathies (Conway et al., 1998; Galvin et al., 2001; Beyer, 2006; Uversky, 2007; Tsigelny et al., 2008; Oueslati et al., 2010; Taschenberger et al., 2012). While most of the pathogenic mutations promote α-syn aggregation, some mutations including G51D which has the earliest onset and severe symptoms, inhibit α-syn aggregation (Narhi et al., 1999; Rospigliosi et al., 2009; Ono et al., 2011; Khalaf et al., 2014; Rutherford et al., 2014; Rutherford and Giasson, 2015; Lazaro et al., 2016; Kumar et al., 2018). Therefore, factors other than α-syn aggregation probably contribute to the pathogenesis (Villar-Pique et al., 2016). In 2003, Braak proposed a hypothesis that pathological α-syn changes first occur in the peripheral nervous system and olfactory bulb, then ascend to the brainstem and midbrain, and finally spread to the forebrain (Braak et al., 2003). This hypothesis was later supported by clinical evidence that nigral neurons grafted into the striatum of PD patients had Lewy body-like inclusions more than 10 years later (Kordower et al., 2008; Li et al., 2008). Since then, cell-to-cell propagation of α-syn has been demonstrated by numerous *in vitro* and *in vivo* studies (Steiner et al., 2011; Eisbach and Outeiro, 2013; Lashuel et al., 2013; Dehay et al., 2016; Uchihara and Giasson, 2016; Breen et al., 2019). Exogenous α-syn aggregates or recombinant preformed α-syn fibrils (PFFs) have been found to enter neurons and act as “seeds” to promote the aggregation of endogenous α-syn, ultimately leading to its pathological spread (Volpicelli-Daley et al., 2011; Angot et al., 2012; Luk et al., 2012; Holmqvist et al., 2014; Borland and Vilhardt, 2017; Rutherford et al., 2017; Bieri et al., 2018; Rey et al., 2018; Henderson et al., 2019). This cell-to-cell transmission property of α-syn prompted us to investigate whether pathogenic mutations modulate the propagation and cytotoxicity of α-syn and whether these changes correlate with the clinical characteristics.

The transfer of α-syn requires secretion from host cells and uptake by recipient cells (Costanzo and Zurzolo, 2013; Dunning et al., 2013; George et al., 2013; Hijaz and Volpicelli-Daley, 2020), and several pathogenic mutations (H50Q, G51D, and A53T) display enhanced α-syn secretion (Fares et al., 2014; Khalaf et al., 2014; Lazaro et al., 2014; Li et al., 2017). However, it remains to be determined whether other mutants have similar effects and whether they affect other steps during cell-to-cell propagation. The aim of this study was to systematically investigate the effects of pathogenic α-syn mutations on different steps of α-syn spread. We generated 8 pathogenic mutations of human α-syn and investigated their effects using assays in differentiated SH-SY5Y cells [a human neuroblastoma cell line differentiated to express more dopaminergic neuron-like properties (Cheung et al., 2009)], rat cortical neurons and microglia, and the rat substantia nigra (SN). The assays assessed protein expression levels, mRNA levels, secretion, cytotoxicity, aggregation, uptake of PFFs by primary cells, seeding, and acute neuroinflammation in the SN pars compacta (SNpc) after PFF injection. Our results shed light on the understanding of PD pathogenesis and provide insight for intervention.

## Materials and Methods

### Antibodies

The following antibodies were used: anti-α-synuclein monoclonal antibody (BD Transduction Laboratories, San Jose, CA, United States), anti-bovine serum albumin (BSA) monoclonal antibody (Abcam, Cambridge, United Kingdom), anti-β-actin monoclonal antibody (Abcam), anti-α-synuclein (phospho S129) monoclonal antibody (Wako, Tokyo, Japan), anti-tyrosine hydroxylase rabbit antibody (Sigma, Merck KGaA, Darmstadt, Germany), anti-Ibal rabbit antibody (Wako), and anti-C-myc rabbit antibody (Santa Cruz, Dallas, TX, United States).

### Expression and Purification of Recombinant α-Syn

A C-myc tag with a linker (sequence: 5′-GSGS-3′) was introduced into all α-syn expression vectors, resulting in the sequence 5′-EQKLISEEDLGSGS-3′. All C-myc-tagged wild-type (WT) α-syn and mutants were subcloned into either pGEX-4-T-2 for bacterial expression (with an N-terminal tobacco etch virus cleavage site followed by an myc epitope tag, leaving an extra N-terminal glycine upon proteolytic removal of the GST moiety), or pLenti-CMV for lentivirus-mediated expression in differentiated SH-SY5Y cells. The 8 point mutations were introduced by site-directed mutagenesis.

BL21 (DE3) cells transformed with a pGEX-4-T-2 plasmid encoding WT human-Syn or its mutants were freshly grown on ampicillin agar plates. Single colonies were transferred to 5 ml LB medium with 50 μg/ml ampicillin (AppliChem, Darmstadt, Germany) and incubated overnight at 37°C with shaking at 250 rpm. This pre-culture was used to inoculate 500 ml of LB/ampicillin medium and the bacteria were grown to an optical density of 0.7–0.8 (measured at 600 nm) before the induction of protein expression with 0.1 mM isopropyl-D-thiogalactopyranoside (Sigma) for 4 h at room temperature. Bacteria were harvested by centrifugation at 8,000 *g* at 4°C.

Cell pellets were re-suspended in 0.1 M phosphate-buffered saline (PBS) buffer (pH 7.5) containing 1 mM EDTA and 1 mM phenylmethylsulfonyl fluoride, 0.5 mg/ml lysozyme, 2 U/ml DNAse1, and an EDTA-free protease inhibitor mixture (Roche) on ice, and ultrasonicated with an output power of 12 W applied in 5-s pulses between 5-s pauses for a total ultrasonication time of 5 min. Cell debris was pelleted by centrifugation at 15,000 *g* for 30 min at 4°C. Proteins were affinity-purified using glutathione Sepharose bead incubation (GE Healthcare) with supernatant overnight at 4°C, followed by cleavage with thrombin (10 U/mg protein, Sigma) for 16 h at 4°C. Purified proteins were concentrated with centrifugal filter units (Millipore) and verified by Coomassie staining and immunoblotting. The concentrations of samples were quantified by Coomassie staining and bicinchoninic acid protein quantification.

### α-Syn Fibril (PFF) Preparation

Purified recombinant α-syn monomer (2 mg/ml for aggregation experiments and 5 mg/ml for injection experiments) was incubated in 20 mM Tris buffer, pH 7.4, containing 0.1% NaN_3_ in a shaking incubator at 1,000 rpm for 7 days at 37°C. All PFFs were identified by thioflavin-T, circular dichroism, and transmission electron microscopy (TEM) and sonicated for 60 s before use. The endotoxin test was performed using the Bioendo GC Endotoxin Test Kit (Gel Clot Assay) according to the manufacturer’s protocol (Bioendo; cat no# EC32545).

### Thioflavin-T Assay

α-Syn aggregation was continuously monitored using Thioflavin-T (Th-T) fluorescence. Protein samples (5 μM final concentration, 50 μl volume) were added into a 96-well plate and incubated with 10 μM Th-T (1:1) in PBS (10 μM, pH 7.4) for 15 min at room temperature. The fluorescence intensity was read using a Spectra Max i3 Multi-Mode Microplate Detection Platform (Molecular Devices) at room temperature. The excitation and emission wavelengths were set at 450 and 485 nm, respectively. The calculation of growth rate was based on previous reports (Evans et al., 1995; Narhi et al., 1999).

### Circular Dichroism (CD) Measurements

Protein samples were analyzed using a Chirascan plus CD spectrometer (Applied Photophysics) at room temperature. Ten microliters of incubated sample was diluted into 190 μl PBS (20 mM, pH 7.4) to a final concentration of 5 μM (0.1 μg/μl). CD spectra were recorded in the range 190–250 nm and we used a 1.0 mm optical path-length quartz cuvette to investigate the secondary structure of α-syn and its mutations. Data points were acquired every 0.2 nm in continuous scanning mode at 50 nm/min with a digital integration time of 2 s and a bandwidth of 1 nm. Processed spectra were obtained by subtracting a blank control (20 mM PBS, pH 7.4) from the protein spectrum. And the Secondary Structure Estimation software of Pro-Data Viewer was used to smooth the curve.

### Transmission Electron Microscopy

The PFF samples (1:5 in deionized ddH_2_O) were applied to formvar-coated copper grids, negatively stained with 1% (w/v) uranyl acetate, and then washed with deionized water. A Philips CM electron microscope (Philips, Eindhoven, Netherlands) at 100 kV was used to collect transmission images.

### Differentiation of SH-SY5Y Cells and Lentivirus Infection

Following the methods reported by Cheung et al. (2009), SH-SY5Y cells from the American Type Culture Collection were differentiated using retinoic acid (RA). In brief, the cells were maintained in Dulbecco’s modified Eagle’s medium (DMEM)/F12 (Hyclone, United States) supplemented with 10% fetal bovine serum (FBS; Hyclone) and 0.1% penicillin and streptomycin (Nacalai Tesque, Japan). After 1 day, they were sub-cultured into 6-well plates (Thermo, United States) at a density of 1 × 10^5^/cm^2^. The cells were then exposed to 10 μM RA (Sigma, United States) in DMEM/F12 containing 2% FBS to differentiate for 5 days. The differentiated SH-SY5Y cells were then infected with lentivirus (MOI 1:5; HeYuan Biotechnology Co., Ltd.) for α-syn expression. After 24 h, the culture medium was completely replaced with F-12 medium containing 10% FBS, 1% penicillin (50 U/ml) and streptomycin (50 mg/ml), and 10 μM RA. Then the cells were continuously cultured for 5 days with complete medium replacement every 2 days.

### Cell Lysis and Concentration of Conditioned Medium

Cells were washed with ice-cold PBS and lysed on ice for 30 min with RIPA buffer (50 mM Tris, 150 mM NaCl, 1% Triton X-100, 1% sodium deoxycholate, 0.1% SDS, 0.1 M EDTA, pH 7.4) in the presence of protease inhibitors. The lysates were centrifuged at 14,000 *g* for 15 min at 4°C, and the supernatants were collected for Western blot analysis.

Conditioned medium was collected and centrifuged at 4°C at 10,000 *g* for 20 min to remove dead cells and debris and the supernatant collected for ELISA (alpha-Synuclein Human ELISA Kit from Invitrogen Thermo Fisher, Carlsbad, CA, United States) or concentrated using 3 kD centrifugal filter units (Millipore) for Western blot.

### Primary Neuronal Cultures and PFF Uptake

Cultures of cortical neurons from Sprague-Dawley rats (embryonic day 18) were prepared as described previously (Beaudoin et al., 2012). Rat pup cortex was dissected in ice-cold D-Hanks buffer (Invitrogen) and collected in a centrifuge tube. The cortical tissue was dissociated by trypsinization (2 ml 0.125% trypsin and 20 U DNAse1 for 15 min at 37°C). The same volume of plating medium [DMEM (Gibco, Waltham, MA, United States) containing 5 g/L glucose and 10% FBS] was added to inhibit trypsinization. Then the tissue was pipetted in plating medium and centrifuged briefly to collect neuronal cells. The cells were re-suspended in plating medium and plated in 6- or 96-well plates coated with poly-D-lysine (1:100; BD Biosciences) at a density of 1 × 10^5^/cm^2^. The plating medium was replaced with growth medium [Neurobasal medium with 2% B-27 supplement and 0.5 mM L-Glutamax-1 (all from Invitrogen)] 4 h after plating. Half of the culture medium was exchanged with growth medium every 3 days. Neuronal cultures at 10–12 days *in vitro* (DIV) were incubated with 1 μM of sonicated WT or mutant PFF for 24 h, 2 days, or 4 days before experiments. For analysis of intracellular α-syn level, cells were washed twice with 10 mM HCl and 150 mM NaCl for 2 min, followed by 2 ml PBS for 10 min, to remove fibrils adhering to the cell surface.

### Primary Microglial Cultures and PFF Uptake

Primary microglial cultures were prepared from Sprague–Dawley rat pups on post-natal days 0–1. Microglia were acquired from primary mixed culture as described previously (Du et al., 2017). Briefly, the brain was removed from each newborn pup and the meninges stripped off in sterile ice-cold D-Hanks (in mM: 137.93 NaCl, 5.33 KCl, 0.44 KH_2_PO_4_, 0.34 Na_2_HPO_4_⋅12H_2_O, 10.00 HEPES or NaHCO_3_, and 5.56 D-glucose, pH 7.4). The tissue was dissociated by repeated aspiration, and the mixed cells were cultured with DMEM/F-12 nutrient mixture (Gibco) containing 10% FBS, 100 U/ml penicillin, and 0.1 mg/ml streptomycin. Microglial cells were separated after 14 days using the “shaking off” method. For Western blotting, microglia were plated at 2 × 10^4^ cells/well in 24-well plates. For reactive oxygen species (ROS) and lactate dehydrogenase (LDH) assays, microglia were plated at 5 × 10^3^ cells/well in 96-well plates. Microglia were allowed to adhere for 24 h before incubation with 1 μM sonicated WT or mutant PFFs for 2 or 24 h before experiments. For analysis of intracellular α-syn levels, cells were washed twice with 10 mM HCl and 150 mM NaCl for 2 min, followed by 2 ml PBS for 10 min, to remove fibrils adhering to the cell surface.

### Cell Viability Assay

Cell viability was evaluated using the protocol described in the MTS [3-(4,5-dimethylthiazol-2-yl)-5-(3-carboxymethoxyphenyl)-2-(4-sulfophenyl)-2H-tetrazolium] assay kit (Abcam). The absorbance was read in a Spectra Max i3 Multi-Mode Microplate Detection Platform (Molecular Devices) at 490 nm.

### Reactive Oxygen Species Assay

The intracellular ROS levels in primary cortical neurons and microglia were measured using 2, 7-dichlorofluorescein diacetate (DCFH-DA), a fluorogenic and plasma membrane-permeant dye, as described previously with slight modification (Delic et al., 2017; Sashourpour et al., 2017). Briefly, primary cortical neurons or microglia were seeded into 96-well plates at a density of 10^5^/cm^2^. On 12 DIV, cells were treated with 2 μM PFFs of α-syn for 2 days. After treatment, the cells were washed with DMEM (without FBS and phenol red; Gibco) and incubated with 10 μM DCFH-DA at 37°C for 30 min, then washed with 0.01 M PBS (pH 7.4) to remove excess DCFH-DA. Then the plates were read with a Spectra Max i3 Multi-Mode Microplate Detection Platform (Molecular Devices) and fluorescence intensity was measured using 488 nm excitation and 530 nm emission.

### PFF Injection Into Rat Substantia Nigra Pars Compacta (SNpc)

For stereotaxic injection, male Sprague-Dawley rats (2–3 months old) were anesthetized intraperitoneally with 2% pentobarbital sodium (0.3 ml/100 g body weight). Two microliters of BSA or sonicated α-syn PFFs (5 mg/ml) was injected into the right SNpc (coordinates: anteroposterior 5.8 mm, mediolateral 2.3 mm, dorsoventral –7.45 mm) at 0.2 μl/min. After injection, the needle was left in place for 5 min before removal and the rats were sacrificed 24 h later.

### Immunofluorescence Analysis

Anesthetized rats were perfused with 300–400 mL of 0.9% sterile normal saline at room temperature, followed by 300–400 mL of ice-cold 4% paraformaldehyde (PFA) in 0.01 M PBS. Brains were post-fixed in 4% PFA for at least 24 h and gradient-dehydrated in 20 and 30% sucrose in 0.1 M PBS. Then, coronal sections were cut at 50 μm on a freezing microtome (Leica).

The sections were treated with 0.3% Triton X-100 in PBS for 30 min, then endogenous peroxidase activity was quenched with 3% hydrogen peroxide in PBS for 30 min. The sections were washed 3 times in PBS and blocked with 2% FBS for 1 h at room temperature. Primary antibodies were diluted in the blocking solution and applied overnight at 4°C. Secondary antibodies conjugated to Alexa Fluor 488 or 594 fluorophores (Invitrogen) were applied for 2 h at room temperature in the dark. Nuclei were stained in Vectashield mounting medium with DAPI (Vector Laboratories). Confocal microscopy was performed with a Leica TCS SP8 confocal microscope under 20 × or 63 × objective magnification.

### Real-Time Quantitative Reverse Transcription-PCR (qRT-PCR)

Total RNA was isolated from cells using TRIzol reagent (Life Invitrogen) and quantified using a NanoDrop 2000 spectrophotometer (Thermo Scientific). Then, the total RNA was converted to cDNA using SYBR Green qPCR Master Mix (Thermo Fisher, United States) according to the manufacturer’s protocol. cDNA was amplified by real-time PCR using the MX3000P Real-Time PCR System (Applied Stratagene Co.). qRT-PCR was used to quantify the expression levels of mRNAs encoding α-syn, tumor necrosis factor-alpha (TNF-α), interleukin (IL)-6, IL-1β, inducible nitric oxide synthase (iNOS), poly (ADP-ribose) polymerase (PARP), and glyceraldehyde-3-phosphate dehydrogenase (GAPDH). The specific rat gene primers were from AuGCT Biotechnology (Beijing, China). qRT-PCR reactions were performed as follows: 1 cycle of 50°C for 5 min and 95°C for 30 s, and 40 cycles of 95°C for 5 s and 60°C for 30 s. Melting analysis was used to confirm the specificity of amplicons. The levels of gene expression were normalized to GAPDH mRNA using the comparative CT method.

The following primers were used:

**Table:** 

Gene symbol	Gene name	Sequence of primers (5′–3′)
α-syn	α-Synuclein	F: GGCTGAGAAGACCAAAGAGCA R: CTGCTCCCTCCACTGTCTTC
IL-6	Interleukin 6	F: CCTACCCCAACTTCCAATGCT R: GGTCTTGGTCCTTAGCCACT
IL-1β	Interleukin 1 beta	F: AGGCTGACAGACCCCAAAAG R: CTCCACGGGCAAGACATAGG
TNF-α	Tumor necrosis factor alpha	F: CCCCCATTACTCTGACCCCT R: CCCAGAGCCACAATTCCCTT
iNOS	Nitric oxide synthase 2	F: GAAGTTCAGCAACAACCCCAC R: CCCTGACCATCTCGGGTGC
PARP	Poly (ADP-ribose) polymerase	F: CGACAAGGAGAGCAGGTATGG R: GTAGTTCGCACTTTTGGACACC
GAPDH	Glyceraldehyde-3-phosphate dehydrogenase	F: TGTGAACGGATTTGGCCGTA R: TGAACTTGCCGTGGGTAGAG
		

### Statistical Analysis

All data shown are the mean ± SEM, and were analyzed using one-way analysis of variance (ANOVA) followed by Dunnett’s test compared to WT α-syn. A *p*-value < 0.05 was regarded as significant and “n” refers to the number of cultures or rats used in each group.

### Ethics Statement

All animal procedures were approved by the Institutional Animal Care and Use Committee of Capital Medical University, Beijing, China.

## Results

### Pathogenic Mutations Increase α-Syn Secretion in Differentiated SH-SY5Y Cells

**FIGURE 1:**
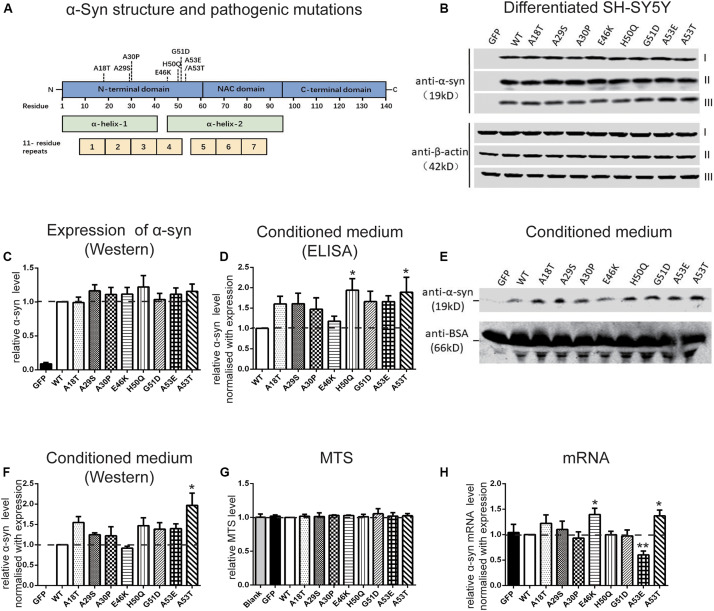
Pathogenic mutations increase α-syn secretion in differentiated SH-SY5Y cells. **(A)** Schematic of pathogenic mutations of α-syn. **(B,C)** Immunoblots **(B)** and analysis **(C)** of cell lysates (*n* = 7 independent cultures, results of three experiments are shown). **(D–F)** Immunoblots (n = 4 independent cultures) and ELISA analysis (*n* = 7 independent cultures) of conditioned medium. **(G,H)** Normalized MTS levels **(G)** and mRNA levels quantified by qRT-PCR (H) (*n* = 4 independent cultures). Mean ± SEM; *P < 0.05, **P < 0.01, one-way ANOVA followed by Dunnett’s test compared to WT.

To investigate the regulation of α-syn cell-to-cell transmission by pathogenic mutations, we first assessed their effects on secretion. The WT human α-syn sequence and the 8 pathogenic mutants, A18T, A29S, A30P, E46K, H50Q, G51D, A53E, and A53T, all of which lie in the N-terminal domain of the α-syn protein (Figure 1A), were inserted into a lentivirus expression vector. Differentiated SH-SY5Y cells were infected with lentivirus expressing WT or mutant α-syn and the conditioned medium was collected for the measurement of α-syn release. Since the level of α-syn secretion is correlated with its expression (Lee et al., 2005), we adjusted the amount of lentivirus to obtain similar expression levels of WT and mutant α-syn in SH-SY5Y cells (Figures 1B,C). The extracellular α-syn in conditioned medium was quantified by both ELISA assays (Figure 1D) and Western blots (Figures 1E,F). Western blots revealed a main band at the molecular weight of the α-syn monomer, showing that most of the secreted α-syn existed in that form. After normalization to their expression level, we found that the H50Q and A53T mutations significantly increased the secretion of α-syn while the other mutations tended to increase compared to WT α-syn in both assays (Figures 1D,F). The increased α-syn secretion was not due to cell lysis caused by mutant expression since cell viability, assayed by MTS reduction, remained unchanged (Figure 1G). This result is consistent with previous reports on the H50Q, G51D, and A53T mutants (Fares et al., 2014; Khalaf et al., 2014; Li et al., 2017).

We also used qRT-PCR to assess the mRNA levels when WT and mutant α-syn were expressed at similar protein levels in differentiated SH-SY5Y cells. The mRNA concentration was normalized to the results of expression levels by Western blot (Figure 1H). The mRNA levels of the E46K and A53T mutants were significantly higher, while that of A53E was significantly lower, than that of the WT. No significant differences were found between the other mutations and the WT.

### Effect of Pathogenic Mutations on α-Syn Fibril Aggregation *in vitro*

**FIGURE 2:**
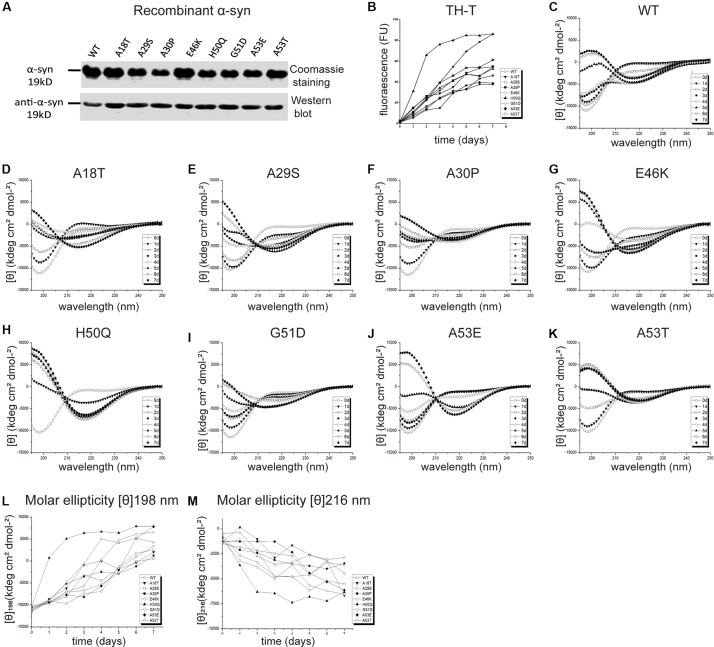
Effects of pathogenic mutations on α-syn aggregation *in vitro*. **(A)** Coomassie staining and immunoblots of recombinant human α-syn (WT) and pathogenic mutants. **(B)** Thioflavin-T (Th-T) binding assays (*n* = 3 independent experiments). **(C–M)** Secondary structure dynamics of α-syn (*n* = 3 independent experiments). **(C–K)** CD spectra of WT α-syn **(C)**, A18T **(D)**, A29S **(E)**, A30P **(F)**, E46K **(G)**, H50Q **(H)**, G51D **(I)**, A53E **(J)**, and A53T **(K)** acquired at the start of incubation, day 0 (∘), and after days 1 (▼), 2 (□), 3 (■), 4 (◆), 5 (▲), 6 (▽), and 7 (◆). **(L,M)** Molar ellipticity [θ]198 nm **(L)** and [θ]216 nm **(M)** with time.

*In vivo* studies of α-syn propagation have shown that microinjection of PFFs efficiently initiates a progressive Parkinson-like neurodegeneration while α-syn monomers have no effect (Masuda-Suzukake et al., 2013; Tarutani et al., 2016; Chung et al., 2019). Furthermore, PFFs trigger a sustained and progressive loss of dopamine neurons compared to oligomeric species (Taschenberger et al., 2012; Froula et al., 2019), although both are neurotoxic (Bousset et al., 2013; Chen et al., 2015). Therefore, we chose PFFs to study the effects of mutants on cellular uptake and cytotoxicity. Recombinant human WT and pathogenic mutant α-syn proteins were purified (Figure 2A) and incubated to generate PFFs. The aggregation kinetics, structural dynamics, and fibril morphology were characterized by thioflavin-T (Th-T), circular dichroism (CD), and TEM, respectively.

Th-T produces a characteristic increase of fluorescence at 480–490 nm upon β-sheet binding (Biancalana and Koide, 2010; Lee et al., 2011; Ono et al., 2011) and was used for quantitative analysis of the fibril aggregation process (Figure 2B). To investigate the aggregation kinetics of the WT and pathogenic mutants, we analyzed the growth rate and maximum intensity (Table 1) and found that the A18T, A29S, A30P, E46K, H50Q, and A53T mutants accelerated α-syn aggregation compared with the WT α-syn, while on the other hand, G51D and A53E decelerated fibril formation. These results are in good agreement with previous reports (Narhi et al., 1999; Rospigliosi et al., 2009; Ono et al., 2011; Khalaf et al., 2014; Rutherford et al., 2014; Rutherford and Giasson, 2015; Lazaro et al., 2016; Kumar et al., 2018). Among all the pathogenic mutations, H50Q promoted and G51D decelerated aggregation most strongly. We found that A30P accelerated α-syn aggregation and increased β-structure content, which agrees with some previous reports (Narhi et al., 1999; Li et al., 2001), but differs from others (Conway et al., 2000; Lazaro et al., 2014). This discrepancy is probably due to an enhanced oligomerization rate early but a decreased fibril-formation rate later by the A30P mutant (Conway et al., 2000; Lazaro et al., 2014), as Th-T can bind oligomers, protofibrils, and fibrils (Lindgren et al., 2005).

**TABLE 1:** Kinetics of α-syn aggregation.

Samples	Thioflavin-T		Circular dichroism		Transmission electron microscopy
		
	Growth rate^a^ (FU/day)	Maximum intensity^b^ (FU)	t_1/2_[θ]198^c^ (∼days)	t_1/2_[θ]216^c^ (∼days)	Diameter (nm)
WT	7.2	46.3	3.7	2.2	11.31 ± 0.12
A18T	10.0	55	2.5	1.8	11.44 ± 0.13
A29S	10.7	61.3	3.1	2.0	11.81 ± 0.12
A30P	9.6	54.5	2.0	3.0	10.01 ± 0.11
E46K	13.4	86.7	2.0	1.5	11.65 ± 0.12
H50Q	25.7	88	0.8	1.1	12.15 ± 0.11
G51D	6.3	37.9	4.0	4.1	9.98 ± 0.12
A53E	6.7	40.2	5.3	4.5	9.11 ± 0.10
A53T	9.6	53.3	2.5	1.8	11.15 ± 0.10

*^*a*^Growth rate determined by line-fitting to the pseudo-linear segment of the ascending portion of the fluorescence progress curve. ^*b*^Determined by visual inspection (FU, fluorescence units). ^*c*^([θ]t – [θ]min)/([θ]max – [θ]min) = 1/2, where molar ellipticity [θ] is measured at 198 nm or 216 nm, [θ]t is molar ellipticity at time t, [θ]max is maximal ellipticity, and [θ]min is minimum ellipticity.*

To assess the dynamics of secondary structure, we used CD spectra to monitor β-sheet filament formation, which was revealed by a negative peak at [θ]216 nm and a positive peak at [θ]198 nm (Figures 2C–K). All of the WT and pathogenic α-syn mutants displayed changes in the secondary structure of amyloid formation during days 0–7. We found that A18T, A29S, E46K, H50Q, and A53T accelerated α-syn aggregation compared with the WT at all data points. On the other hand, G51D and A53E decelerated fibril formation. These results are in agreement with previous reports (Fares et al., 2014; Rutherford et al., 2014; Lazaro et al., 2016). Interestingly, A30P showed faster changes at [θ]198 nm during the first 4 days but slowed afterward compared to the WT, supporting our conclusion above. To allow comparison of the kinetics among WT and pathogenic mutants, we determined the half-times of [θ]198 nm and [θ]216 nm (Table 1 and Figures 2L,M). Strikingly, the H50Q mutant showed the fastest kinetics in both Th-T and CD experiments. The overall activity of fibril formation was in the order H50Q > E46K > A53T > A18T > A29S > WT > A30P > A53E > G51D.

**FIGURE 3:**
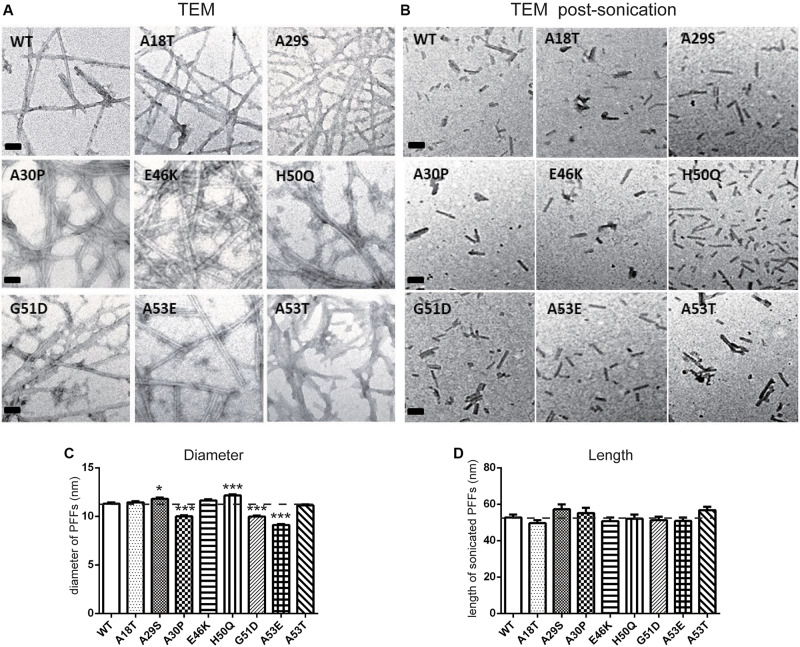
Transmission electron microscopy analysis of PFFs. **(A,B)** TEM images of negatively stained PFFs before **(A)** and after sonication **(B)** (scale bars, 50 nm). **(C,D)** Diameter **(C)** and length analysis **(D)** of PFFs (mean ± SEM; *P < 0.05, ***P < 0.001 compared to WT, one-way ANOVA followed by Dunnett’s test compared to WT; *n* = 300 in 3 independent experiments).

We used TEM to directly visualize the PFFs of WT and pathogenic mutants. After incubation for 7 days, all PFFs formed long amyloid fibrils with a highly ordered structure (Figures 3A,C; diameters of PFFs listed in Table 1). This is consistent with previous reports on the WT and the A30P, E46K, H50Q, G51D, A53E, and A53T mutations (Li et al., 2002; Fredenburg et al., 2007; Ono et al., 2011; Rutherford et al., 2014; Rutherford and Giasson, 2015), while A18T and A29S have not been reported. Interestingly, the diameters of A30P, G51D, and A53E fibrils were significantly smaller than that of the WT, and all of these mutations decelerated the aggregation of α-syn. On the other hand, H50Q fibrils had the greatest diameter. This result suggests that faster aggregation kinetics is correlated with more efficient fibrillization.

### Effect of Pathogenic Mutations on Intracellular α-Syn Level and Cytotoxicity of Extracellular PFF Treatment in Primary Neurons and Microglia

**FIGURE 4:**
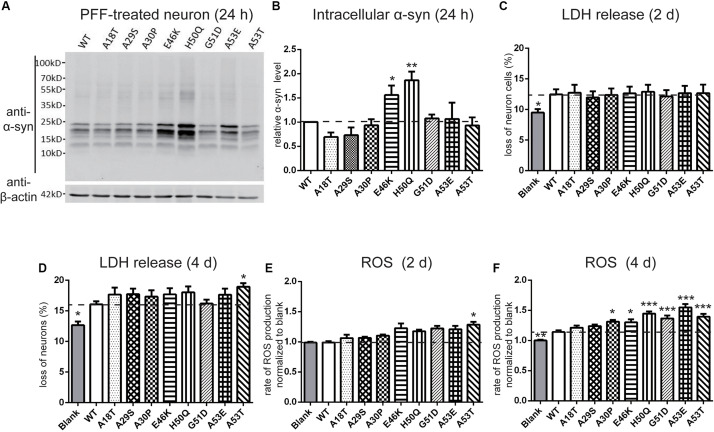
Effects of pathogenic mutations on intracellular α-syn levels and cytotoxicity of extracellular PFF treatment in primary cortical neurons. Neurons were treated with 1 μM sonicated WT or pathogenic mutant PFFs at 37°C for 24 h. **(A,B)** Immunoblots and analysis showing levels of intracellular α-syn. **(C,D)** Percentages of cell death measured by LDH release after 2 and 4 days treatment with sonicated PFFs. **(E,F)** ROS assays after PFF treatment. Mean ± SEM; *P < 0.05, **P < 0.01, ***P < 0.001, one-way ANOVA followed by Dunnett’s test compared to WT; *n* = 3 independent experiments.

Next, we set out to assess the effects of extracellular PFF on cultured neurons and microglia. As the short fragments of PFFs are the most effective triggers of the prion-like spread of α-syn *in vivo* (Tarutani et al., 2016; Froula et al., 2019), rat primary cortical neurons were incubated with 1 μM of sonicated WT and mutant PFFs of similar lengths (Masaracchia et al., 2018) (calculated based on the initial concentration of monomeric α-syn) (Figures 3B,D). The average lengths of WT and pathogenic mutations were < 60 nm, in agreement with the reported optimal fibril length (Tarutani et al., 2016; Abdelmotilib et al., 2017). Western blot analysis was used to quantify the intracellular α-syn level after neurons were acid-stripped to remove PFFs adhering to the surface and lysed. Intracellular levels in E46K and H50Q were significantly higher than in the WT and other mutants (Figures 4A,B). Taking into account factors such as degradation during the incubation times, the data showed that the fibril uptake and/or degradation of these two mutations differed from the WT and the other mutations.

To assess the cytotoxicity of the WT and mutant PFFs to primary neurons, we measured LDH release after 2 and 4 days of PFF treatment (Figures 4C,D). The death rate of neurons treated with PFFs was significantly higher than that of the blank control at both time points, consistent with previous reports on the cytotoxicity of WT PFFs (Mahul-Mellier et al., 2015; Mao et al., 2016). Compared with the WT, the pathogenic mutations did not significantly alter cell death after 2 days of treatment, but after 4 days, the A53T mutant showed significantly stronger cytotoxicity (Figure 4D). We then used a ROS assay to evaluate the oxidative insult. Only A53T showed significantly higher ROS production after 2 days of PFF treatment. However, the ROS production of neural cells treated with mutant PFFs was higher in general, except for A18T and A29S, compared with the WT after 4 days of treatment (Figures 4E,F).

**FIGURE 5:**
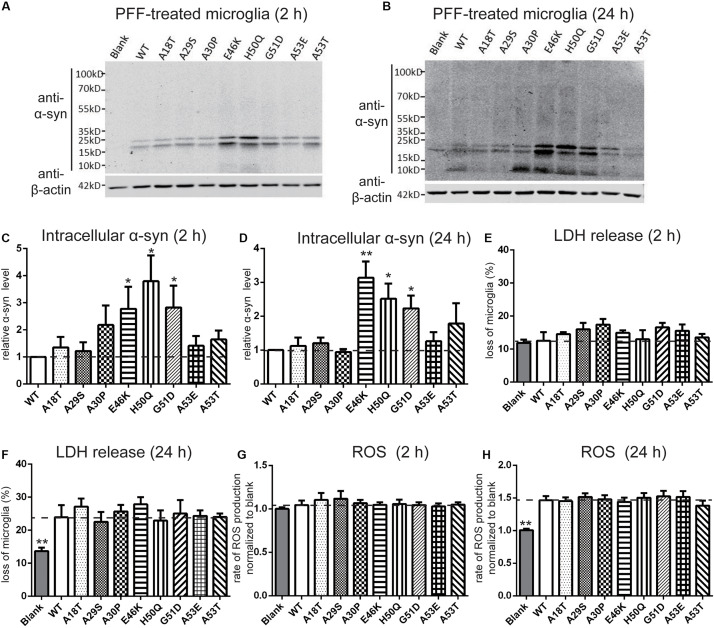
Effects of pathogenic mutations on the intracellular α-syn levels and cytotoxicity of extracellular PFF treatment in primary microglia. Microglia were treated with 1 μM sonicated WT or pathogenic mutant PFFs at 37°C for 2 h **(A,C,E,G)** and 24 h **(B,D,F,H)**. **(A–D)** Immunoblots and analysis of intracellular α-syn. **(E,F)** Cell viability assessed by LDH release. **(G,H)** Oxidative stress assessed by ROS assays. Mean ± SEM; *P < 0.05, **P < 0.01, one-way ANOVA followed by Dunnett’s test compared to WT; *n* = 3 independent experiments.

It has been reported that extracellular PFFs are most efficiently taken up by microglia (Dos-Santos-Pereira et al., 2018; Feng et al., 2019), the activation of which in the SN plays an important role in the progression of PD (Zhang et al., 2005; Harms et al., 2017; Qiao et al., 2017). To evaluate the effect of the pathogenic mutations on intracellular levels of α-syn, primary microglial cells were isolated from the rat brain and treated with sonicated PFFs for 2 and 24 h. Similar to primary neurons, the intracellular levels in the E46K and H50Q mutations as well as G51D significantly increased compared with the WT and the other mutations at 2 and 24 h (Figures 5A–D). To assay the toxicity of WT and pathogenic mutant PFFs in microglia, we measured LDH release after 2 and 24 h of treatment. The death rates did not significantly differ between cells treated with PFFs and controls at 2 h (Figure 5E). However, the death rate increased significantly at 24 h, with no significant difference between the WT and pathogenic mutants (Figure 5F). This result suggested similar cytotoxicity to microglia of WT and mutant PFFs. We also measured ROS production under the same conditions and found that it was similar in microglia treated with PFFs and controls after 2 h of treatment (Figure 5G). After 24 h, the PFFs induced more ROS production in microglia than in controls (Figure 5H), but there was no significant difference between the WT and pathogenic mutants.

### Mutant PFFs Trigger Stronger Seeding and Acute Inflammatory Responses in the SNpc

**FIGURE 6:**
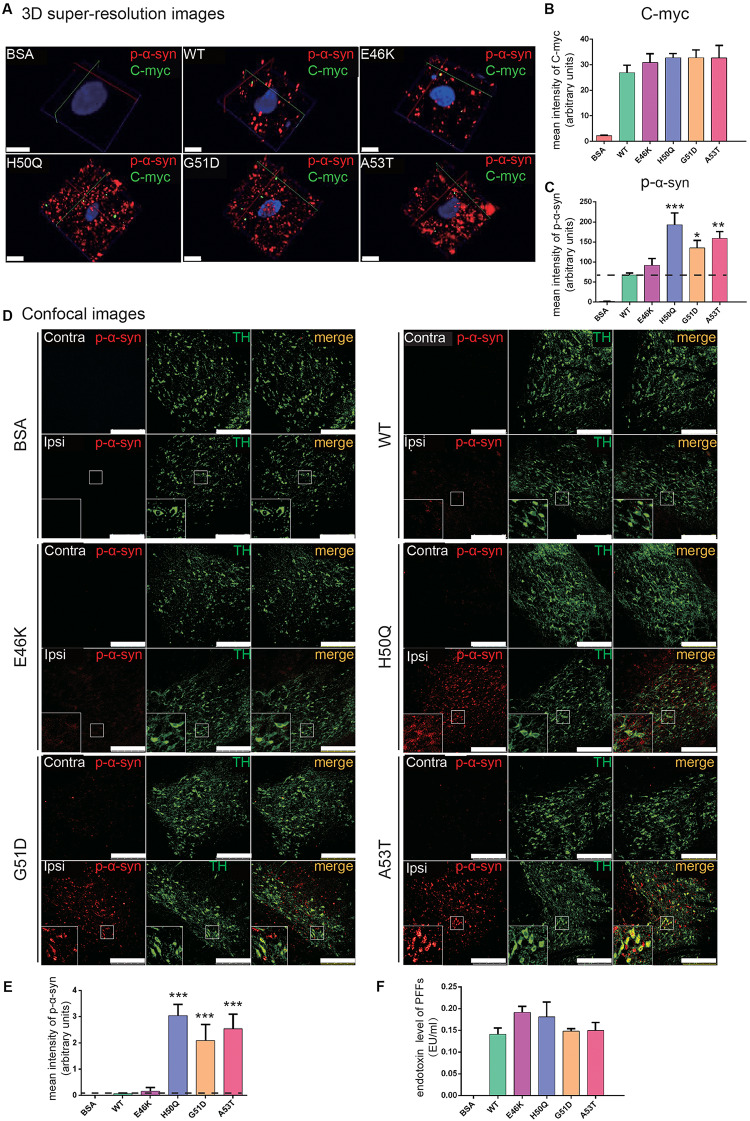
PFFs induce phosphorylation of endogenous α-syn in dopaminergic neurons. **(A)** 3D super-resolution images of internalized PFFs (C-myc, green) co-stained with endogenous p-α-syn (red) 24 h after injection (scale bars, 5 μm). **(B,C)** Mean intensity of C-myc **(B)** and p-α-syn **(C)** in 3D super-resolution images. **(D)** Confocal images of TH (green) co-stained with p-α-syn (red) (scale bars, 250 μm). **(E)** Mean intensity of p-α-syn in confocal images as in **(D)**. **(F)** Endotoxin levels in microinjected samples. Mean ± SEM; *P < 0.05, **P < 0.01, ***P < 0.001, one-way ANOVA followed by Dunnett’s test compared to WT; *n* = 6 animals.

To study the *in vivo* effects of mutant PFFs, we selected four pathogenic mutations, E46K, H50Q, G51D, and A53T, which caused increased intracellular α-syn levels or cytotoxicity. Stereotaxic injections of WT or mutant PFFs into the right SNpc of adult male Sprague-Dawley rats were performed and brain sections were immunostained 24 h later (the same time point as the cellular uptake assays). BSA served as a negative control. Since we purified recombinant α-syn from bacteria, we first made sure that the endotoxin levels of the PFFs were < 0.5 endotoxin units (EU)/ml to prevent triggering of inflammatory responses or cytotoxicity *in vivo* (Polinski et al., 2018). The endotoxin levels of all four samples were <0.2 EU/ml (Figure 6F). WT and mutant PFFs all induced the phosphorylation of endogenous α-syn at Ser129 in dopaminergic neurons in the ipsilateral SNpc 24 h after injection while no signal was detected in the BSA controls (Figures 6A,D). Three-dimensional super-resolution microscopy revealed that exogenous PFFs (visualized by antibody against C-myc) were taken up by neurons, accompanied by the phosphorylation of endogenous α-syn (Figure 6A). Quantification of individual dopamine neurons showed similar C-myc signals in the WT and mutants (Figure 6B) but enhanced phospho-α-syn (p-α-syn) staining in the H50Q, G51D, and A53T groups (Figure 6C), suggesting that these mutants have a stronger seeding capacity. Few C-myc puncta were detected inside cells. It is possible that the C-myc levels were under-represented since exogenous seeds may have been surrounded by endogenous α-syn aggregates and became inaccessible to myc antibodies.

When we examined the overall p-α-syn levels in the SNpc with confocal microscopy, we found that H50Q, G51D, and A53T triggered a significantly higher mean intensity of p-α-syn than the WT 24 h post-injection (Figures 6D,E). This result is consistent with a previous report on G51D PFF injection in mouse SNpc at 12 or 24 weeks post-injection (Hayakawa et al., 2019). P-α-syn was localized in tyrosine hydroxylase-positive dopamine neurons (Figure 6D, insets). On the other hand, E46K had a much weaker effect, similar to the WT.

**FIGURE 7:**
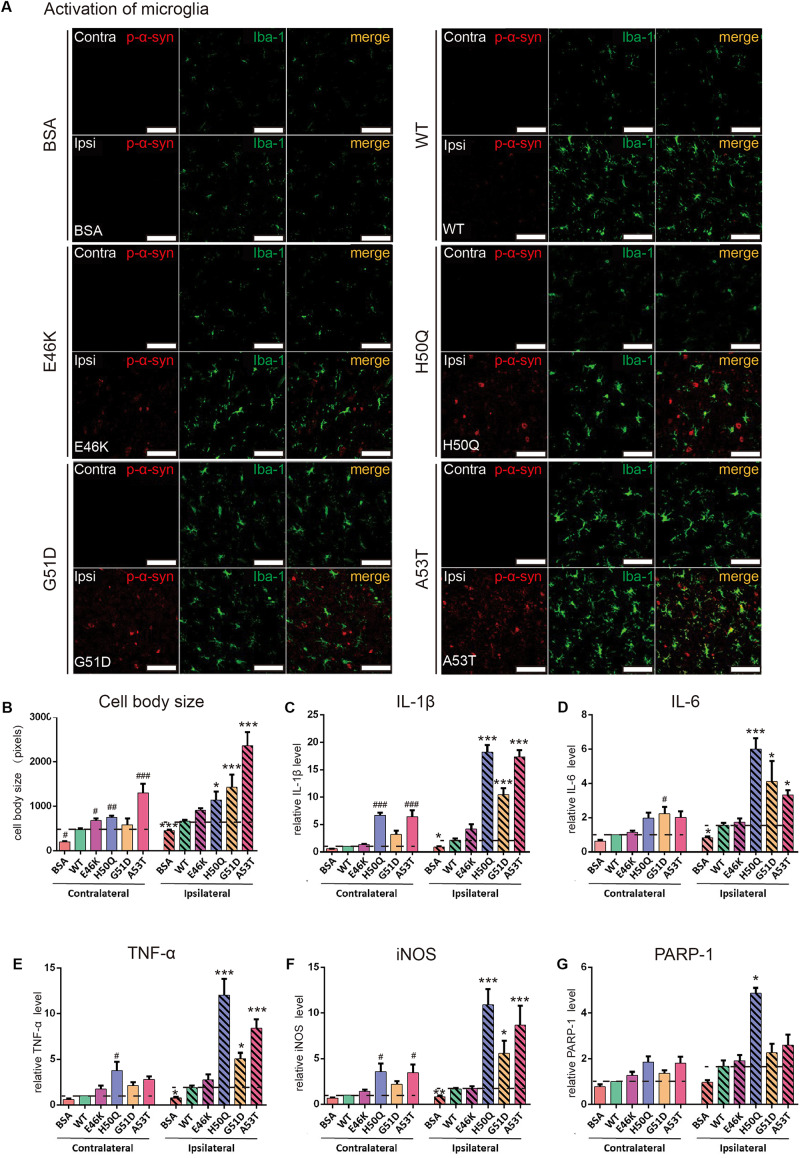
WT and pathogenic mutant PFFs activate microglial cells. **(A)** Confocal images of microglia (Iba1, green) co-stained with p-α-syn (red) 24 h after injection (scale bars, 100 μm. **(B)** Cell body size of microglia. **(C–G)** mRNA levels of IL-1β **(C)**, IL-6 **(D)**, TNF-α **(E)**, iNOS **(F)**, and PARP-1 **(G)** assessed by qRT-PCR. Mean ± SEM; contralateral:^#^*P* < 0.05,^##^*P* < 0.01, ^###^*P* < 0.001 compared to WT; ipsilateral: *P < 0.05, **P < 0.01, ***P < 0.001, one-way ANOVA followed by Dunnett’s test compared to WT; *n* = 6 animals.

Activation of microglia was also found in the SNpc (Figure 7). When the cell body sizes of microglia on the ipsilateral and contralateral sides were measured, we found that the extent of microglial activation by H50Q, G51D, and A53T PFFs, but not E46K PFFs, was significantly greater than that of the WT on the injected side, and A53T caused the strongest activation. Meanwhile, A53T also induced the strongest activation of microglia on the contralateral side (Figures 7A,B). To further investigate the inflammatory response induced by PFFs, we quantified the mRNA levels of inflammatory cytokines (IL-1β, IL-6, TNF-α, iNOS, and PARP-1) by qRT-PCR (Figures 7C–G). The H50Q, G51D, and A53T mutants notably increased the level of the inflammatory response, H50Q having the most severe effect. Conversely, there was no significant difference between the E46K mutant and the WT, similar to their effects on p-α-syn (Figure 6E). Taken together, mutant PFFs induced stronger phosphorylation of endogenous α-syn and neuroinflammation.

## Discussion

The cell-to-cell transmission of α-syn aggregates between neurons has provided important clues for elucidating the pathogenesis of PD. Although pathogenic mutations of α-syn are very rare, they have been directly linked to disease progression and severity and have helped reveal the molecular mechanisms underlying neurodegeneration (Devine et al., 2011). In this study, we systematically investigated the modulating effects of pathogenic mutations on the cell-to-cell transmission of α-syn (Table 2) and revealed the following:

**TABLE 2:** Effects of pathogenic mutants.

					PFF treatment of neuron			PFF treatment of microglia			Microinjection in SNpc	
							
Mutation	Onset age	Secretion	mRNA	Aggregation	Intracellular α -syn	Toxicity	Oxidative stress	Intracellular α -syn	Toxicity	Oxidative stress	P-alpha-synuclein	Neuro-inflammation
A18T	Late			+							NA	NA
A29S	Late			+							NA	NA
A30P	Late			+ /−			+				NA	NA
E46K	Early		+ +	+ +	+ + +		+	+ + +				
H50Q	Early	+ + +		+ + +	+ + +		+ +	+ + +			+ + +	+ + +
G51D	Early			−−−			+	+ + +			+ + +	+ + +
A53E	Early		−−	−			+ +				NA	NA
A53T	Early	+ + +	+ +	+ +		+	+				+ + +	+ + +

*Compared to WT: “+” <20% increase; “+ +” 20–50% increase; “+ + +” >50% increase. “−“<20% decrease; “−−” 20–50% decrease; “−−−“>50% decrease.*

(1) Pathogenic mutations of α-syn tended to show increased secretion levels overall, with H50Q and A53T enhancing secretion significantly compared to the WT.
(2) While A30P, G51D, and A53E delayed α-syn aggregation, the other pathogenic mutations promoted it. Among the 8 mutations, G51D delayed, while H50Q promoted, aggregation most strongly.
(3) Compared to the WT, E46K, and H50Q significantly increased the intracellular α-syn level upon PFF treatment in both primary neurons and microglia, and G51D increased the level in microglia, while the other mutations had no effect.
(4) When the PFFs of α-syn were microinjected into the SNpc, H50Q, G51D, and A53T but not E46K acutely induced extensive α-syn phosphorylation and neuroinflammation *in vivo* compared to the WT.

Taken together, pathogenic mutations facilitate the prion-like spread of α-syn at discrete steps in the process.

The secretion of α-syn prompts the paracrine role of extracellular α-syn in brain homeostasis, leading to a cascade of events including neuronal degeneration under pathological conditions (Vekrellis et al., 2011; Marques and Outeiro, 2012). Several pathogenic mutations have been found to promote the release of α-syn (Fares et al., 2014; Khalaf et al., 2014; Li et al., 2017). Our data showed that pathogenic mutations generally increased α-syn secretion compared to the WT. This is consistent with previous reports that the release of α-syn is upregulated under conditions of cellular stress due to the accumulation of misfolded/damaged proteins (Kordower et al., 2008; Christensen et al., 2016). Secretion of α-syn may serve as a double-edged sword. On one hand, neurons release damaged α-syn, which may be cleared by extracellular proteolytic enzymes (Stefanis et al., 2019), reducing its intracellular toxicity. On the other hand, extracellular α-syn may be taken up by neighboring glia and neurons, triggering neuroinflammation and cell-to-cell transmission to promote the spread of pathology (Stefanis, 2012; Dunning et al., 2013; Recasens and Dehay, 2014). Therefore, it is plausible that pathogenic mutations promote α-syn propagation through increased secretion, which may partly account for the early onset of PD. The secretion of α-syn is calcium-dependent and brefeldin A-insensitive, suggesting a non-classical vesicular exocytosis pathway (Lee et al., 2005; Emmanouilidou et al., 2010; Jang et al., 2010), so future studies are needed to understand how these mutations affect this pathway. In addition, whether the released α-syn is toxic to cells and whether seeding-competent synuclein oligomers occur in our conditioned media deserves future study using assays such as PMCA (protein misfolding cyclic amplification) (Wang et al., 2018). The mRNA levels of mutations that had similar protein expression levels suggested that E46K and A53T degraded faster while A53E degraded slower than the WT. Previous reports have shown that E46K and A53T enhance α-syn phosphorylation at Ser-129 in mammalian cell lines (Mbefo et al., 2015), while p-α-syn is preferentially degraded by proteasomes (Machiya et al., 2010). It is possible that the different degradation rates found here are due to the phosphorylation states. Future studies can examine the kinetics and mechanisms of degradation by pulse-chase experiments.

α-Syn PFFs are capable of cell-to-cell transmission and initiate PD-like neurodegeneration in animal models (Polinski et al., 2018). Multiple pathways of PFF uptake by neurons and microglia have been proposed, such as receptor-mediated endocytosis, phagocytosis, and tunneling nanotube formation (Sung et al., 2001; Desplats et al., 2009; Aulic et al., 2017; Du et al., 2017; Bieri et al., 2018). In our study, the E46K and H50Q mutants generated higher intracellular levels of α-syn PFFs in primary neurons and the E46K, H50Q, and G51D mutations caused higher levels in microglia, while the other mutations were similar to the WT. This shows that pathogenic mutations also differentially modulate the cellular uptake and/or degradation of α-syn aggregates, and E46K and H50Q aggregates may spread from cell to cell more efficiently. It should be noted that our method could not distinguish external from internal fibrils, although we applied acid stripping to remove most of the fibrils adhering to the cell surface. A membrane-impermeable trypan blue quenching assay with fluorescently labeled α-syn PFFs would be better to address this question (Karpowicz et al., 2017). In addition, all the α-syn PFFs induced cytotoxicity and oxidative stress in neurons and microglia, and the mutants had a greater effect in general. The degree of toxicity caused by the different mutations was not simply correlated with their intracellular levels, suggesting that each PFF has intrinsic properties that induce a cellular response.

As PD patients carrying E46K, H50Q, G51D, or A53T mutations have early onset of the disease, the propagation of α-syn pathology probably starts before the processes of aging. The human SN is highly vulnerable to α-syn pathology and the level of α-syn in the SNpc increases with age (Li et al., 2004; Chu and Kordower, 2007; Xuan et al., 2011). To better simulate the cell-to-cell transmission of mutant PFFs at an early stage of PD pathogenesis, we used adult WT rats for unilateral SNpc injection of α-syn PFFs, and assessed p-α-syn along with neuroinflammation on the ipsilateral side 24 h after injection. Interestingly, the H50Q, G51D, and A53T PFFs evoked a p-α-syn signal > 10-fold that of the WT while the signal of E46K was similar to the WT. This finding is consistent with a previous report on PFF injection into the hippocampus of transgenic mice (the M20 line, overexpressing human α-syn) using E46K, H50Q, G51D, and A53E fibrils, after which they assessed p-α-syn (Rutherford et al., 2017). While they examined the effects of PFF injection after 2 or 4 months, we found efficient seeding as early as 24 h after injection, suggesting a convenient assay for assessing PFF seeding activity *in vivo*. However, it remains to be determined whether or not the endogenous p-α-syn induced by PFFs is soluble or insoluble, since soluble p-α-syn is targeted to proteasomes and lysosomes for degradation (Machiya et al., 2010; Liu et al., 2015). Among E46K, H50Q, G51D, and A53T, we found that E46K was inefficient in seeding α-syn pathology, as the myc signal indicated similar PFF levels inside neurons from the WT and the mutant. We suspect that the early onset of E46K is mainly caused by its high cell-autonomous toxicity as a soluble species (Inigo-Marco et al., 2017). The *in vivo* uptake of PFFs differed from that in cultured neurons where E46K and H50Q increased PFF uptake compared to WT and the other mutants. This discrepancy may be due to the different detection methods used, each with its own limitations, or the different molecular mechanisms involved in cultured cells and *in vivo*. More in-depth analysis is necessary to elucidate whether cellular models can recapitulate each step of the *in vivo* propagation of α-syn. The neuroinflammatory responses to PFFs were similar to those of p-α-syn, where H50Q induced the most robust reaction. Furthermore, the side contralateral to the mutant PFF injection also reacted in cytokine, iNOS, and PARP-1 expression, revealing a rapidly spreading neuroinflammatory response. Our results showed that intracerebral injection of H50Q, G51D, or A53T PFFs can create robust animal models of PD. A recent study has suggested that there is insufficient clinical evidence for pathogenicity of H50Q in PD (Blauwendraat et al., 2018). However, residue H50 has been shown to protect α-syn from aggregation *in vitro* (Chi et al., 2014) and here we also showed unique pathological features of H50Q. Therefore, we suggest that this mutant deserves further investigation.

The prion-like transmission of α-syn involves multiple steps, including secretion from host cells, extracellular spread, uptake by receptor cells, and seeding in receptor cells. Secretion and endocytosis are both crucial cellular processes. Seeding is quite efficient in the presence of α-syn aggregates as we showed here, most likely because α-syn is an abundant cytosolic protein. Therefore, we suggest that removing extracellular α-syn aggregates may be a promising intervention strategy for α-syn transfer, as their abundance in cerebrospinal fluid is very low and extracellular α-syn monomers do not appear to be pathogenic (Danzer et al., 2007; Tarutani et al., 2016; Gustafsson et al., 2017; Sardi et al., 2018; Gao et al., 2019; Hijaz and Volpicelli-Daley, 2020).

We conclude that pathogenic mutations enhance the prion-like spread of α-syn at discrete steps. There may not be a simple correlation between their modulation and the disease onset and progression. Multiple factors seem to be involved. Revealing the effects of PD-causing mutations on α-syn behavior benefit not only our understanding of the pathogenesis, but also the future development of therapeutic intervention.

## Data Availability Statement

The raw data supporting the conclusions of this article will be made available by the authors, without undue reservation, to any qualified researcher.

## Ethics Statement

The animal study was reviewed and approved by the Institutional Animal Care and Use Committee of Capital Medical University, Beijing, China.

## Author Contributions

YG, XZ, FL, SY, YW, and CD performed the experiments. YG and XZ analyzed the data. CZ conceived the project. CZ, RL, and XC supervised the project. YG, XZ, CZ, and RL wrote the manuscript. All authors read and approved the final manuscript.

## Conflict of Interest

The authors declare that the research was conducted in the absence of any commercial or financial relationships that could be construed as a potential conflict of interest.

## Abbreviations

α-syn: α-synuclein
CD: Circular Dichroism
DCFH-DA, 2: 7-dichlorofluorescein diacetate
DMEM: Dulbecco’s modified Eagle’s medium
EU: endotoxin units
IL: interleukin
iNOS: inducible nitric oxide synthase
LDH: lactate dehydrogenase
p- α-syn: phospho- α-syn
PARP: poly (ADP-ribose) polymerase
PD: Parkinson’s disease
PFA: paraformaldehyde
PFFs: α-syn fibrils
PFFs: preformed α-syn fibrils
qRT-PCR: real-time quantitative reverse transcription-PCR
RA: retinoic acid
ROS: reactive oxygen species
SN: substantia nigra
SNpc: substantia nigra pars compacta
TEM: transmission electron microscopy
Th-T: Thioflavin-T
TNF- α: tumor necrosis factor-alpha
WT: wild-type.
